# Management of temporo-mandibular joint ankylosis using different surgical approaches

**DOI:** 10.6026/973206300191359

**Published:** 2023-12-31

**Authors:** Bibhu Prasad Mishra, Satyajit Sahu, Arup Ratan Das, Asutosh Pradhan, Priyankesh, Shekhar Gupta

**Affiliations:** 1 Department of Oral and Maxillofacial Surgery, Hi-tech Dental College and Hospital, Bhubaneshwar, Odisha, India; 2 Department of Oral and Maxillofacial Surgery, Hi-tech Dental College and Hospital, Bhubaneswar, Odisha, India; 3 Department of Oral and Maxillofacial Surgery Hi-tech Dental College and Hospital Bhubaneswar, Odisha, India; 4 Department of Oral and Maxillofacial Surgery, Hi-tech Dental College and Hospital, Bhubaneshwar, Odisha, India; 5 Department of Dentistry, Manipal Tata Medical College, Manipal Academy of Higher Education, Karnataka, India; 6 Department of Prosthodontic Dental Sciences, College of Dentistry, Jazan University, Jazan 45142, Saudi Arabia

**Keywords:** Surgical approaches, TMJ ankylosis, distraction osteogenesis gap arthroplasty, interpositional arthroplasty

## Abstract

Comparison of gap arthroplasty (GAP), interpositional arthroplasty (IAP) and distraction osteogenesis (DO) simultaneous with interpositional arthroplasty (DO+IAP) in management of TMJ ankylosis is of interest to dentists. The study comprised 36 individuals with TMJ ankylosis, 16 of whom were female and 20 of whom were male. Both prior to and following surgery, the maximum inter-incisal opening (MIO) and facial pattern were noted. The postoperative MIO was 33.23 ± 1.23mm, 35.24 ± 1.11mm and 38.24 ± 1.34mm in GAP, IAP and DO+IAP respectively. Data is statistically significant with high MIO observed in DO+ IAP technique and low MIO in GAP technique (p < 0.005). In addition to lengthening the mandible, concurrently processed interpositional arthroplasty alongside DO for TMJ ankylosis corrects gross asymmetry of the face, occlusal mal-alignment, midline change, and creates room for previously un-erupted teeth to emerge.

## Background:

A medical problem known as temporo-mandibular joint (TMJ) ankylosis impairs the ability to masticate and carry out daily activities [1-2]. It can cause constrained chewing capacity, swallowing along with speech difficulties, as well as impaired oral as well as nutritional health [3- 4]. Patients may exhibit a variety of manifestations according to the kind and degree of engagement, the age at which the condition first appeared, and the time frame of the ankylosis [5- 6]. This leads to a change in the standard potential for growth during development, which in turn causes facial asymmetry, micrognathia of mandible, obstructive breathing while sleeping, and a decrease in the regular functional boosts required for the maturation of the maxillofacial complex as a whole [7-8]. Management for TMJ ankylosis requires a timely diagnosis, suitable medical treatment, alongside suitable rehabilitation [9- 10].

Recurrence is still the main issue despite the fact that a number of treatments, such as TMJ reconstruction, interpositional arthroplasty (IAP), gap arthroplasty (GAP) have been presented [11-12]. Nevertheless, no published unanimous agreement has been attained regarding these approaches. The significant likelihood of recurrence that comes with GAP with no interpositional substance has led numerous surgeons to slowly give up the procedure [13- 14]. A common procedure called IAP places alloplastic or autogenous material in between the temporal bone's surface and ramus of mandible [15-16]. The most popular material is the temporalis myofascial flap (TMF), which has the advantages of being simple to harvest and having a reduced risk of resorption. But this flap also comes with issues like trismus, chronic headaches, and donor site complications [17-18].

Traditionally, autogenous transplantation was the preferred reconstructive choice for management of ankylosis once the integrated joint and micrognathia of mandible were treated with distinct surgeries [19,20]. The best requirements for a graft were that it should be able to take on a comparable shape and structure, be sufficiently sturdy to hold it in place, be able to respond to forces exerted by mastication, and be sufficiently dynamic to withstand ongoing physiologic damage [21,22]. However, the optimal reconstruction ought to tackle the afflicted fossa and the mandibular portion in addition to restoring full form and function and, if applicable, preventing and treating any secondary facial deformities [23,24].

An ensuring idea for this condton is distraction osteogenesis (DO), which has been applied to the regrowth of all affixed soft tissue parts and the building up of a fresh autologous bone which appears like a condyle, along with the creation of an intervening layer composed of fibrous tissue that acts as a pseudo disc [8-14]. Through lengthening of the soft tissue together bone and creating new bone with an analogous embryonic origin and nature, the regenerative approach known as osteo-distraction has found significant use in the treatment of obstructive sleep apnea and cosmetic revision of facial deformities [25,26,27]. The physiologic surroundings and forces that are provided by the concurrent lengthening of soft and hard tissues further aid in remodelling and are thus viewed as a glimmer of hope for finding the perfect cure for this joint integration condition [29,30]. Therefore, it is of interest to generate evidence towards the role of DO in TMJa and develop an algorithm for use of DO in TMJa. Hence, we compare DO+IAP, GAP and IAP in management of TMJ ankylosis.

## Methods and Materials:

The study comprised 36 individuals with TMJ ankylosis, 16 of whom were female and 20 of whom were male. The patients' ages ranged from between the ages of six years old and 21 years old, with an average age of ten years, and the study was conducted between 2018 and 2023. Following GAP, DO and costochondrol graft re-organization of the joint and was explained to all individuals who were able to regain growth. Patients who refused to have their joints rebuilt were not incorporated in the study. Twelve patients each were placed into three distinct categories. GAP was done on patients in category 1, IAP was done on patients in category 2 and DO+IAP was carried out in category 3 (Table 1).

The main complaint among all the patients was limited mouth opening for a long time. For verification of the diagnosis, computed tomography (CT) radiographic examination was employed. The medical condition was then assessed. In order to exclude infections of the upper respiratory tract, a paediatrician evaluation was necessary, and wellness for surgical treatment of TMJa under general anesthesia was established. For every patient, a prior to operation temporal shave was done. All patients received antibiotic prophylaxis prior to surgery in accordance with surgical concepts. All of the patients had trouble receiving general anesthesia due to their reduced mouth opening. Every patient gave permission for an emergency tracheostomy and difficult intubation. Of the patients, twelve underwent blind nasal intubation and the remaining twenty-four underwent fiberoptic intubation. Every patient had a postoperative CT scan performed.

## Assessment:

Radiological as well as clinical examinations were part of the evaluations. Both prior to and following surgery, the high inter-incisal opening (MIO) and facial pattern were noted. Cone beam computed tomography (CBCT) was the primary radiological examination utilised, and all patients consented to preoperative as well as postoperative CT scans.

## Surgical technique:

Following intubation, the individual was covered and outlined with povidone iodine. Vaseline ribbon gauze was stuffed into the external auditory canal. The design of the incision was made where the facial skin meets the ear helix. According to Bramley's description, the incision was made superiorly from the highest point of the helix. A vasoconstrictor was subcutaneously injected after the incision was marked, and the incision was created via the subcutaneous tissues as well as the skin all the way to the temporalis fascia's depth. The periosteal elevator was used for blunt dissection along the whole length of the incision, and a Langenback retractor was used to retract the flap anteriorly. Antero superiorly, just in front of the tragus, an incision has been cut via the outermost layer of the temporalis fascia, starting from the base of the zygomatic arch. At this point, the fat globules seemed visible. The pointy tip of the periosteal elevator was introduced into the fascial incision and dissection was then performed deeply to the superficial portion of the temporal fascia, about 1 cm below the arch. The intervening tissues were then substantially released posteriorly along the direction of the initial incision. The flap pulled back anteriorly at this plane, exposing the ankylotic mass. Burs were used to remove the mass at first, and then a chisel was used to finish the job until the table showed a high mouth opening of between 30 and 35 mm.

## Technique for harvesting temporalis myofascial flap:

Methylene blue was used to create an outline of the flap with sufficient dimensions that centered inferiorly on the fascia. The flap had been extended as much superiorly as was required to provide the right amount of length for the joint lining. The muscle and fascia were dissected to the appropriate depth. Merely the outer layer of the muscle had been removed to ensure a sufficient thickness for the joint lining. The flap was set up between glenoid fossa and ramus and extended to zygomatic arch. It was fastened to the arch using 3-0 ethilon.

## Distraction osteogenesis concurrently with IAG:

The tradtonal Al-Kayat and Bramley cut was utilised to reach the TMJ area because it provides a straight path to the ankylosed TMJ porton. To reveal the ankylotic bony portion, the periosteum covering the ramus and zygomatic arch were cut and raised. In order to safeguard the tissues present medially to the condyle, a condylar retractor was inserted. The bone was cut off by making two incisions, one beneath the recognisable zygomatic arch and the other approximately 1.5 cm below it. A straight fissure bur was used for cutting the bone from the posterior border of TMJ to the sigmoid notch or the anterior portion of the coronoid process. The portion of bony mass was then removed with a chisel. A big round bur smoothed out the uneven edges. Considering its close proximity, user-friendliness, the durability from its base relationship, being available at the identical surgical site, and absence of functional or cosmetic complications, a temporalis myofascial flap was raised and placed between the mandible and the zygomatic arch. In addition, we believe it provides bulk and may provide resistance to re-ankylosis, particularly in DO. The identical procedure-an osteotomy-was used in each case to treat DO. This was accomplished by creating an osteotomy in the angle area and, following exposure through a submandibular incision, implanting a bone-borne unidirectional distractor.

Three to five days after surgery, prophylactic antibiotics were administered. Two weeks after the procedure, the patients were placed on a soft diet, and the drainage was eliminated 24 hours after the procedure. In order to restore normal function of the muscles and avoid hypo-mobility brought on by fibrous adhesions, physiotherapy was initiated on days 5-7 following surgery and was maintained for a low of six months. All cases had a 12- to 24-month follow-up period. CT scans were done and MIO as well as facial pattern were compared before and after surgery.

## Statistical analysis:

The software SPSS version 18.0 (SPSS Inc., Chicago, IL, USA) was used to analyse the data. The intergroup enhancement in MIO was found to be significant using the Wilcoxon rank sum test. The significance of the intergroup recurrence rate and facial pattern modifications was assessed using the x2 test. Statistical significance was established at p values ≤ 0.05.

## Results:

The values of preoperative findings like MIO, percentage of study participants with facial asymmetry, number of study participants with unilateral TMJ ankylosis and bilateral ankylosis was comparable in all three study groups were comparable.(Table 1). The MIO in category of GAP, IAP and DO+IAP was 3.55±0.34mm, 3.14± 0.21mm and 3.47± 0.11mm respectively. Facial asymmetry was observed in all study participants in all three categories. 10 (6L+4R), 9 (5L+4R) and 10 (5L+4R) study participants had unilateral TMJ ankylosis in GAP, IAP and DO+IAP respectively (Table 2). The etiology of TMJ ankylosis was trauma at birth, infection, trauma due to fall and unknown in all three categories. The number of study participants with these etiologies was comparable in all three categories (Table 3). The postoperative MIO was 33.23±1.23mm, 35.24±1.11mm and 38.24± 1.34mm in GAP, IAP and DO+ IAP respectively. The findings were statistically significant with high MIO observed in DO+ IAP technique and low MIO in GAP technique (p<0.005). The rate of recurrence was 20.21%, 10.37% and 06.21% in GAP, IAP and DO+ IAP respectively. The rate of recurrence was low in DO+ IAP technique while it was high in GAP technique. The findings were statistically significant (p<0.005). There was change in facial pattern in 70.23%, 12.36% and 81.48% respectively in GAP, IAP and DO+ IAP respectively. The findings were statistically significant with high proportion of change in facial pattern observed in DO+ IAP technique and low proportion in GAP technique (p<0.005) as shown in Table 4. Chronic headache was observed in 13.43%, 09.28% and 2.23% patients in GAP, IAP and DO+ IAP respectively. Post-operative local infections were found in 23.21%, 14.12% and 9.12% patients in GAP, IAP and DO+ IAP respectively. There were no cases of permanent facal palsy in any patient in any category. Post-operative complications were lower in DO+IAP (Table 5).

## Discussion:

Normally, after separate surgeries were performed to treat the mandibular micrognathia and integrated joint, autogenous transplantation was the recommended reconstructive option for managing ankylosis [12-14]. The most ideal criteria for a graft were that it should be able to adopt a similar structure and form, be strong enough to maintain its position, react to forces generated during mastication, and have enough elasticity to endure continuous physiological harm [15-17]. But the ideal reconstruction should address both the affected fossa and the mandibular portion, restore full form and function, and treat and prevent any secondary facial deformities if applicable [18-21].

Distraction osteogenesis (DO) is a promising concept for this condition that has been applied to the regeneration of all attached soft tissue segments, the formation of a new autologous bone that resembles a condyle, and the formation of an intervening layer made of fibrous tissue that functions as a pseudo disc [11-17]. Osteo-distraction is a regenerative approach to bone formation that creates new bone with an analogous embryonic origin and nature by lengthening the soft tissue that surrounds bone. This technique has been effectively used to treat obstructive sleep apnea and correct facial deformities cosmetically [17-23]. There is hope for a cure for this joint integration condition because of the physiologic conditions and forces created by the simultaneous lengthening of soft and hard tissues, which further aid in remodelling [15-21].

A study described a case in which a young patient with grave bony TMJ ankylosis underwent arthroplasty, condylectomy and mandibular lengthening using DO. The patient's mouth opened 35 mm fifteen months after surgery, and there was no sign of ankylosis. They also claimed that the changes of facial asymmetry were satisfactory [12-18]. In 6 young individuals with TMJa and deformity of mandible a previous study used concurrent IAP and DO to rectify the mandibular malformation and achieve adequate mouth opening and cosmetic accomplishments [15-24]. Another study used concurrent IAP and DO in 11 patients to accomplish acceptable mouth opening, raise mandibular length of body of mandible, and fix asymmetry of face [25,29].

While inverted "L" osteotomy alongside bone graft and SSRO alternatives are available. A study additionally dealt with the issue of ankylosis alongside micrognathia in adults and proposed that DO is more appropriate to correct these abnormalities [21- 28]. Another investigator was able to incorporate bone grafting in a genioplasty process to correct the deformity on both sides, but the patient required another procedure to enhance her chin prominence [29- 30]. A group of surgeons has treated seven cases of TMJ ankylosis using a fascia flap along with temporalis muscle, but they haven't dealt with the issue of micrognathia or the complications that come with it [21- 30].

A previous investigation on 55 patients utilising costo-chondral rib grafts revealed that the average rise in opening of the mouth was greater than twenty five mm in thirty two patients, between five mm and twenty-five mm in ten patients, and less than five mm in ten patients [23-27]. In their analysis of four cases, investigators utilising the second metatarsal as a rehabilitative material demonstrated an average rise in opening of the mouth of thirty six mm. It is rare to obtain an average postoperative greatest interincisal opening larger than 35 mm, irrespective of the technique employed [18-26] In his research, investigator discovered that sheep's body weight had increased, with an overall average of two and half kilogram in eighty percent of cases [21-25].

In three of the fourteen cases that examined, investigators found that there was transient facial nerve fragility. In his analysis of seven cases, another study found no complications [7,27]. In his investigation of 23 patients, scientists found that 3 patients experienced transient preauricular paresthesia, 1 patient experienced intraoperative dura exposure, and 2 patients experienced infection [21- 28]. In his analysis of 16 patients, another study found that there were no cases of infection or bleeding, one patient who had permanent facial nerve weaknesses, and three cases where there were temporary facial nerve weaknesses [18-27]. In his analysis of 76 cases, investigators discovered that one patient had an infection and those two patients had transient facial nerve weakness [21- 26].

The benefits of DO outweigh the drawbacks of the various methods for treating TMJ ankylosis with micrognathia, including orthognathic surgery and costochondral grafts. Compared to orthognathic surgery, DO lengthen bones [25- 27]. With DO, the soft tissues are stretched gradually in order to allow them to acclimatize to the bony movements and possibly reduce the recurrence rate compared to traditional orthognathic surgery. Unlike costochondral grafts, which may require graft harvesting due to donor site morbidity, DO does not have this risk. Unpredictable growth is also linked to cardio-chondral grafts [29-30].

## Conclusion:

In addition to lengthening the mandible, concurrently processed interpositional arthroplasty alongside DO for TMJ ankylosis corrects gross asymmetry of the face, occlusal mal-alignment, midline change, and creates room for previously unerupted teeth to emerge. We believe that this approach for TMJ ankylosis assists in reducing the duration of treatment, eliminates the need for another tracheostomy in the event that the fiberoptic intubation is not feasible, and reduces the requirement for another surgery if the operation were to be performed in two stages.

## Figures and Tables

**Table 1:** Distribution of study participants

	**Treatment approach**	**Number of study participants**
Category 1	Gap arthroplasty (GAP)	12
Category 2	Inter positional arthroplasty (IAP)	12
Category 3	Distraction osteogenesis with Interpositional arthroplasty (DO+ IAP)	12

**Table 2:** Preoperative findings

	**Gap Arthoplasty (GAP) (n=12)**	**Inter positional arthroplasty (IAP) (n=12)**	**DO+ IAP (n=12) (n=12)**	**P value**
Preoperative MIO (mean± SD)	3.55±0.34	3.14± 0.21	3.47± 0.11	>0.005
Facial asymmetry (%)	100	100	100	>0.005
Unilateral TMJ ankylosis	10 (6L+4R)	9 (5L+4R)	10 (5L+4R)	>0.005
Bilateral ankylosis	2	3	2	>0.005
L= left side; R= Right side

**Table 3:** Etiology of TMJ ankylosis

	**Gap Arthoplasty (GAP) (n=12)**	**Interpositional arthroplasty (IAP) (n=12)**	**DO+ IAP (n=12)**	**P value**
Trauma at birth	2	3	4	>0.005
Infection	4	3	2	>0.005
Trauma due to fall	2	2	3	>0.005
Unknown	4	4	3	>0.005

**Table 4:** Post-operative outcomes after three surgical procedures

	**Gap Arthoplasty (GAP) (n=12)**	**Interpositional arthroplasty (IAP) (n=12)**	**DO+ IAP (n=12)**	**P value**
(Mean±SD) postoperative MIO (mm)	33.23±1.23	35.24±1.11	38.24± 1.34	<0.005
Recurrences (%)	20.21	10.37	6.21	<0.005
Change in facial pattern (%)	70.23	12.36	81.48	<0.005

**Table 5:** Complications in three surgical approaches

	**Chronic headache (%)**	**Local infection (%)**	**Permanent facial palsy (%)**
Gap Arthroplasty (GAP) (n=12)	13.43	23.21	0
Interpositional arthroplasty (IAP) (n=12)	9.28	14.12	0
DO+ IAP (n=12)	2,23	9.12	0
